# Genome-wide screen for cell growth regulators in fission yeast

**DOI:** 10.1242/jcs.200865

**Published:** 2017-06-15

**Authors:** Louise Weston, Jessica Greenwood, Paul Nurse

**Affiliations:** Cell Cycle Laboratory, The Francis Crick Institute, London NW1 1AT, UK

**Keywords:** Cell growth, TOR, Sck2, Fission yeast, Transcription

## Abstract

Cellular growth control is important for all living organisms, but experimental investigation into this problem is difficult because of the complex range of growth regulatory mechanisms. Here, we have used the fission yeast *Schizosaccharomyces pombe* to identify potential master regulators of growth. At the restrictive temperature, the *S. pombe pat1^ts^ mei4Δ* strain enters the meiotic developmental program, but arrests in meiotic G2 phase as *mei4^+^* is essential for meiotic progression. These cells do not grow, even in an abundance of nutrients. To identify regulators of growth that can reverse this growth arrest, we introduced an ORFeome plasmid library into the *pat1^ts^mei4Δ* strain. Overexpression of eight genes promoted cell growth; two of these were core RNA polymerase subunits, and one was *sck2^+^*, an S6 kinase thought to contribute to TORC1 signalling. Sck2 had the greatest effect on cell growth, and we also show that it significantly increases the cellular transcription rate. These findings indicate, for the first time, that global transcriptional control mediated through S6 kinase signalling is central to cellular growth control.

## Abstract (precis)

**Summary:** Using a novel system to identify cell growth regulators, we have discovered a number of factors that activate growth in cells in which growth is developmentally switched off.

## INTRODUCTION

The regulation of cell growth is important for all living organisms. The highly conserved kinase target of rapamycin (TOR) has been shown to play a major role in cell growth through the coordination of numerous cellular processes in response to stress and nutritional changes (Loewith and Hall, 2011). Although some downstream effectors have been identified in yeast and mammals, it is still unclear how a positive TOR signal is transduced into cell growth regulation, leading to cell mass accumulation. Furthermore, there is a paucity of assays that measure directly cell growth per se; in particular, a system in which cell growth is switched on in an otherwise non-growing cell has been lacking. Establishing such an assay in a system in which TOR is conserved would illuminate the presently unknown strategies used by cells to activate their growth programme.

The rod-shaped fission yeast, *Schizosaccharomyces pombe*, doubles in mass and length during the cell cycle, generating two daughters of equal size after medial division. Newly divided daughter cells grow from only a single end of the cell, until part way through the cell cycle, when the other end of the cell starts to grow. Bipolar growth continues until cells enter mitosis, at which point, the cells are held at a constant length until nuclear division completes and septation initiates. These restraints over cellular growth during different phases of the cell cycle imply that there is global regulation of cellular growth and hint at the existence of cell growth master regulators (Mitchison, 2003; Navarro et al., 2012).

Growth regulation of fission yeast is responsive to nutrient availability. Tor2 is the catalytic component of the TORC1 complex and is essential for growth, whereas Tor1, of the TORC2 complex, regulates growth during stress and starvation responses. Some of the downstream effectors of TOR signalling are known; however, how TORC1 activity leads mechanistically to the regulation of cell growth is not clear. One of the best-characterised downstream effectors of TORC1 is the S6 kinase (S6K1, also known as RPS6KB1, in human and Sch9 in budding yeast), which phosphorylates multiple substrates involved in protein translation, transcription and cellular metabolism (Jorgensen et al., 2004; Urban et al., 2007; Huber et al., 2009; Lee et al., 2009; Wei and Zheng, 2009). Three S6 kinases in *S. pombe*, Sck1, Sck2 and Psk1, are thought to act downstream of TOR signalling, but the specific functions of each kinase are not well understood (Jin et al., 1995; Fujita and Yamamoto, 1998; Nakashima et al., 2012). Deletion of *sck2^+^* renders cells resistant to TORC1 inhibition and leads to a decreased polysome-to-monosome ratio, suggesting an effect on translation; however, whether this effect is direct or indirect is not clear. Sck2 also influences cell size at division, as *sck2^+^* deletion results in smaller cells and overexpression of *sck2^+^* promotes cell lengthening (Rallis et al., 2014).

Nutrient depletion is the trigger for fission yeast to enter the sexual developmental programme, and this process involves both the TOR and MAPK pathways (reviewed in Yanagida et al., 2011; Broach, 2012). When nitrogen is absent, cell growth is turned off, triggering two progressive cell divisions without intervening growth phases to yield small cells arrested in G1. If cells of the opposite mating type are present in the population, cells will sexually differentiate and progress through pre-meiotic S-phase and enter meiotic G2, before undertaking meiosis I and II. Inhibition or inactivation of Tor2 in mitotically dividing cells induces a phenotype reminiscent of nitrogen starvation, where cells divide without growth, and arrest in G1 (Uritani et al., 2006), and Tor2 downregulation is necessary for cells to enter the sexual developmental programme (Alvarez and Moreno, 2006). Deletion of *tor1^+^* renders cells defective in the nitrogen-starvation response, which then leads to a defect in mating (Kawai et al., 2001; Weisman and Choder, 2001).

In fission yeast, meiosis can be triggered experimentally without nutrient depletion by inhibition of the kinase Pat1 (Iino and Yamamoto, 1985; Nurse, 1985). Shifting cells harbouring a temperature-sensitive *pat1* allele, *pat1-114*, to the restrictive temperature leads to the activation of Mei2, an RNA-binding protein essential for premeiotic DNA synthesis, and entry into meiosis I (Yamamoto, 1996). Deletion of *mei4^+^* in *pat1-114* cells prevents entry into meiosis I and II, and causes cells to arrest in meiotic G2 at the restrictive temperature (Borgne et al., 2002). Here, we show that *pat1-114 mei4*Δ cells do not grow in this meiotic G2 arrest, in contrast to cells in mitotic G2 arrest, where cell growth and elongation continues. Importantly, this meiotic downregulation of growth occurs even in the presence of abundant nutrients. This suggests that the interruption of growth that occurs during meiosis is not simply a result of nutrient limitation, but is an actively controlled cellular process. This situation therefore provides a model system in which to identify controls acting on cell growth. Based on this system, we have screened a genetic overexpression library in *pat1-114 mei4*Δ cells undergoing this developmental switch to identify genes able to override the observed downregulation of growth.

## RESULTS

### Developmental growth arrest in fission yeast

**Fig. 1.**
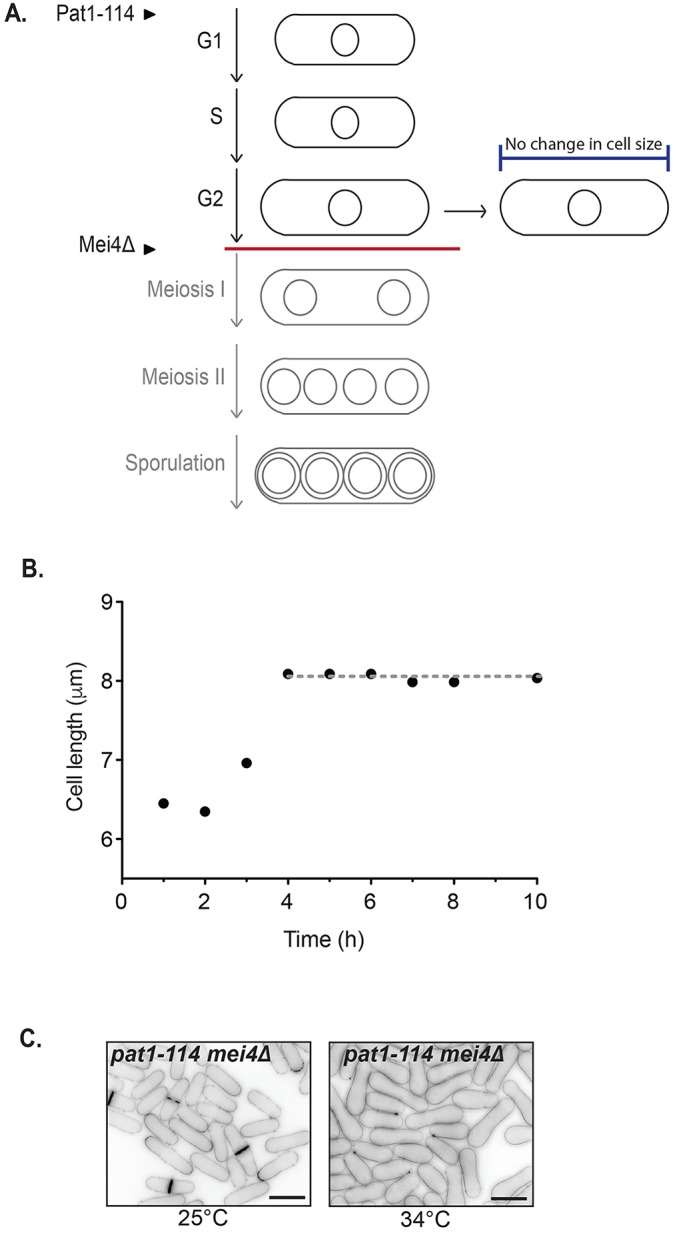
**A system to screen for cell growth regulators.** (A) The *pat1-114* mutant enters the meiotic program upon temperature shift to 34°C. Deletion of *mei4^+^* blocks progression (red line) into meiosis I and II. Cells do not continue to elongate in the meiotic G2 block. (B) *Pat1-114 mei4Δ* cells were synchronised by nitrogen starvation and moved to 34°C at time 0. Cells were measured, and the mean cell length is shown (*n*>30 cells per time point). (C) Cells that initiate the meiotic program interrupt cellular growth, even when supplied with nutrients. *Pat1-114 mei4Δ* cells were grown for 22 h in EMM at 25°C prior to shifting to the restrictive temperature at 34°C (right), or not (left), for 6 h. Scale bars: 10 μm.

Incubating cells harbouring the temperature-sensitive *pat1-114* allele at the restrictive temperature of 34°C drives cells into the meiotic developmental programme. Deletion of *mei4^+^* blocks progression into the meiotic divisions, leading to an arrest in meiotic G2 (Fig. 1A). We examined the cell cycle profile of *pat1-114 mei4Δ* cells that were synchronised by nitrogen starvation prior to meiotic activation. Upon temperature shift, cells exited G1, and by 3 h most were blocked in G2 (Fig. S1A) (Borgne et al., 2002). Average cell length increased from 6.5 μm to 8 μm during the first 4 h after the temperature shift and then remained constant at 8 μm for up to 10 h in the G2 block (Fig. 1B). We repeated the experiment in complete Edinburgh minimal medium (EMM), which contains a nitrogen source, and found that cells attained a cell length of only ∼12 µm (by 6 h at 34°C) and then ceased growth (see cells in Fig. 1C). Cell viability was examined by a colony formation assay, carried out at each time point of the 34°C arrest by plating cells at 25°C. We found that G2 arrested cells showed no decrease in viability up to 6 h after temperature shift although viability did gradually decrease over the following 18 h compared with that seen in wild-type cells (Fig. S1B). These data indicate that the *pat1-114 mei4Δ* strain undergoes a developmental switch-off of cell growth even in the presence of nutrients.

### Genome-wide screen for regulators of cell growth

**Fig. 2.**
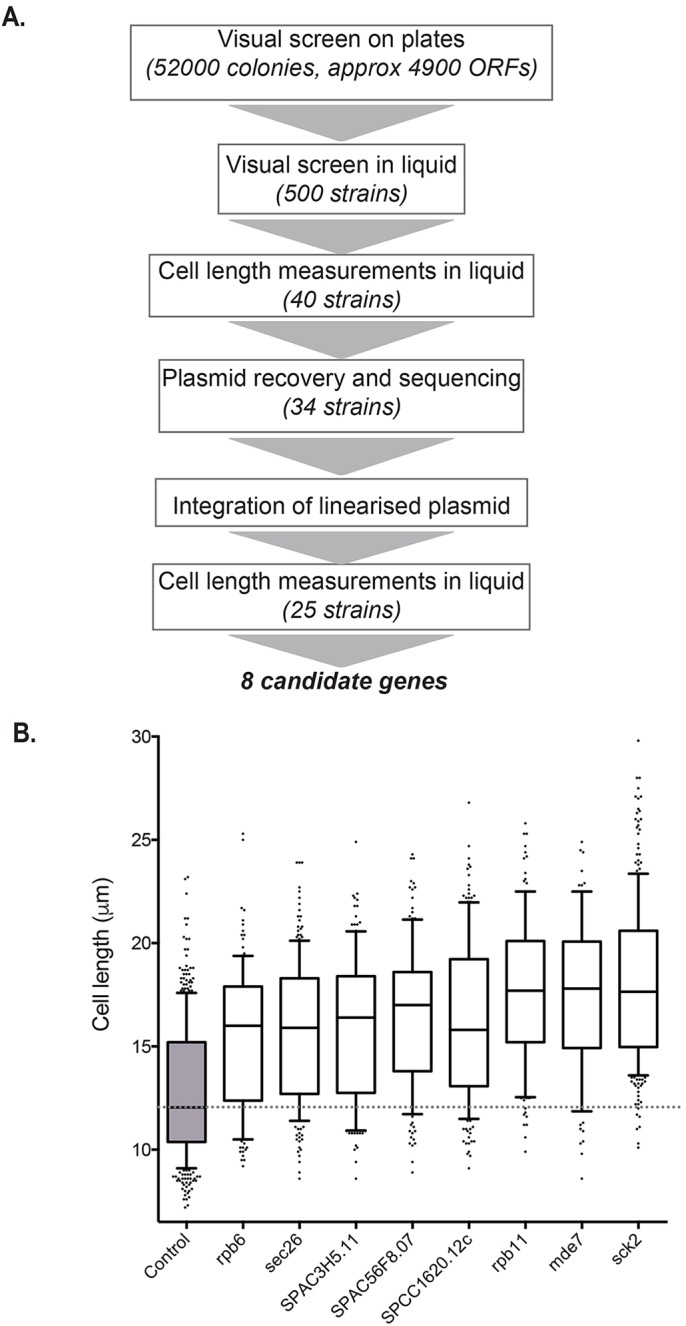
**Identification of genes that promote cell growth.** (A) Schematic of screen to identify genes that, when overexpressed, result in the elongation of cells in meiotic G2 arrest. (B) Cell length measurements for cells of the *pat1-114 mei4::natMX6 ura4 D18 leu1-32* strain with candidate genes integrated at the *leu1* locus after exponential growth in EMM-L for 22 h at 25°C and 6 h further growth at 34°C. Boxes are delimited by the first quartile, median and third quartile, and whiskers show the 10–90th percentiles. Values outside this range are displayed as individual dots. Only strains with >30% median length increase over control are shown. The control strain (A01) harbours a pseudogene in the same vector that is integrated at the *leu1* locus and which has no effect on cell length. More than 100 cells per condition were analysed.

We used this system to carry out a screen to identify genes that can circumvent the growth arrest. We expected that such genes might encode master regulators of cell growth that can override the signalling process that inhibits growth. The Riken ORFeome plasmid library, which covers 96% of fission yeast protein-coding genes and pseudogenes, was used to identify genes that, when overexpressed, could reinitiate growth during the meiotic arrest (Matsuyama et al., 2006). Plasmid pools from the library, harbouring 4910 clones under the thiamine-repressible *nmt1* promoter, were transformed into the *pat1-114 mei4Δ* strain, and transformants were screened. The screening procedure is summarised in Fig. 2A, and consisted of an initial microscopic visual screen, followed by cell length measurements of candidate gene overexpression strains in the meiotic G2 arrest (see Materials and Methods). The screening procedure was carried out at both permissive and restrictive temperatures to rule out genes that cause mitotic cell cycle delay when overexpressed; such strains would be elongated when they entered the meiotic arrest. We identified 40 transformants that exhibited cell size elongation in the *pat1-114 mei4Δ* arrest. The transformed plasmids were recovered from these strains, sequenced and integrated into the *leu1* locus of the *pat1-114 mei4Δ* strain in order to obtain stable expression (Matsuyama et al., 2004). Twenty-five genes were found to cause cell size elongation specifically during the *pat1-114 mei4*Δ meiotic G2 arrest (Table S1; Fig. S2).

### Analysis of putative growth regulators

Of the 25 genes identified, eight caused an increase in cell length of 30% compared to the control strain and were studied further (Fig. 2B). Three of these genes do not currently have experimentally confirmed biological roles in *S. pombe* (Table 1). The five remaining genes include *mde7^+^*, which encodes a RNA-binding protein with a role in meiosis and *sec26^+^*, which encodes a protein involved in vesicle-mediated transport. More informatively, two of the five characterised genes are RNA polymerase subunits, suggesting that transcription could play a role in cell growth reactivation in our system. Additionally, the candidate gene that gave the greatest cell elongation phenotype was *sck2^+^*, which encodes a serine/threonine kinase and is an orthologue of Sch9, the S6 kinase downstream of TOR in budding yeast (Fujita and Yamamoto, 1998).

**Table 1.**
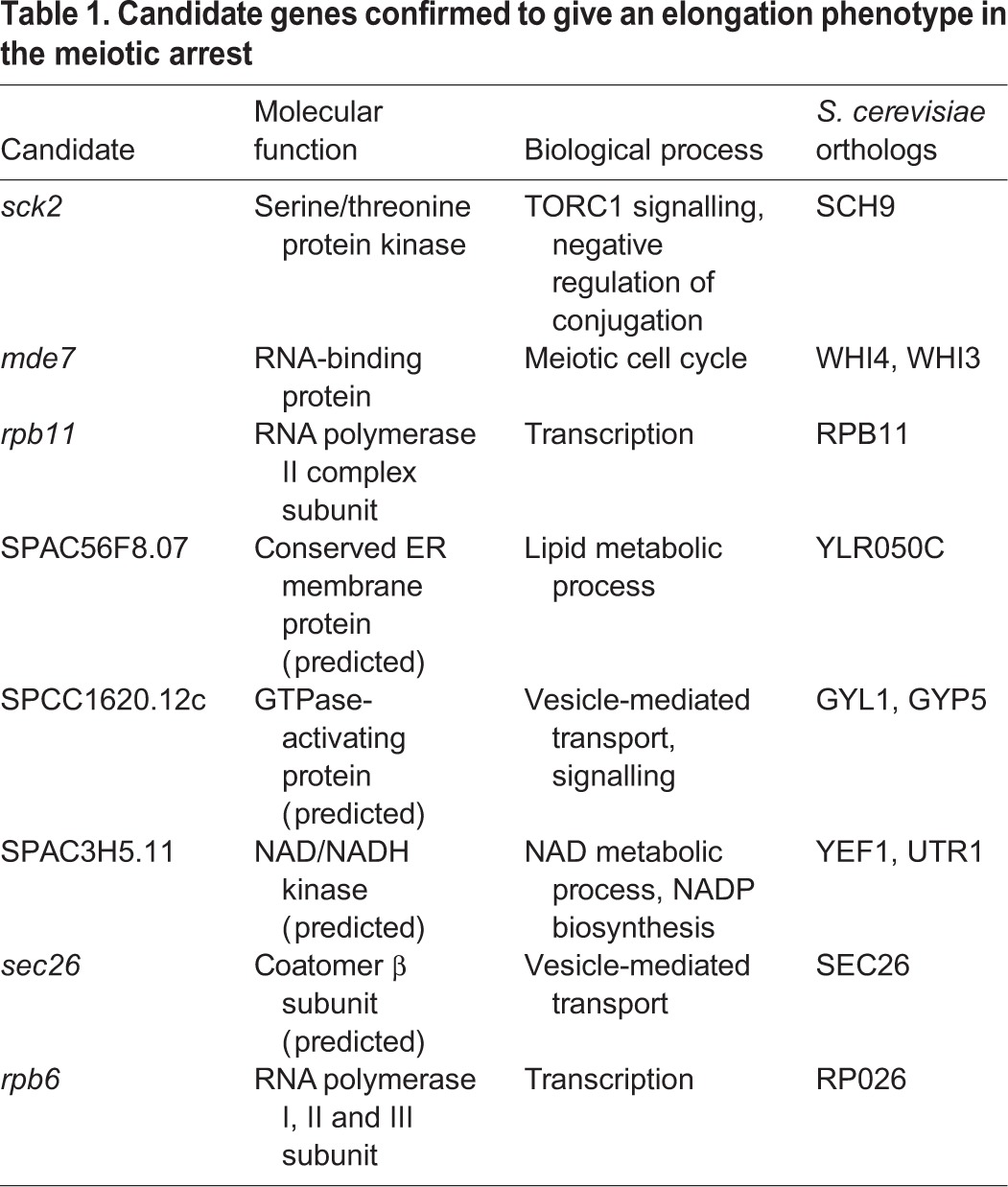
**Candidate genes confirmed to give an elongation phenotype in the meiotic arrest**

### Sck2 – an activator of cell growth and transcription

**Fig. 3.**
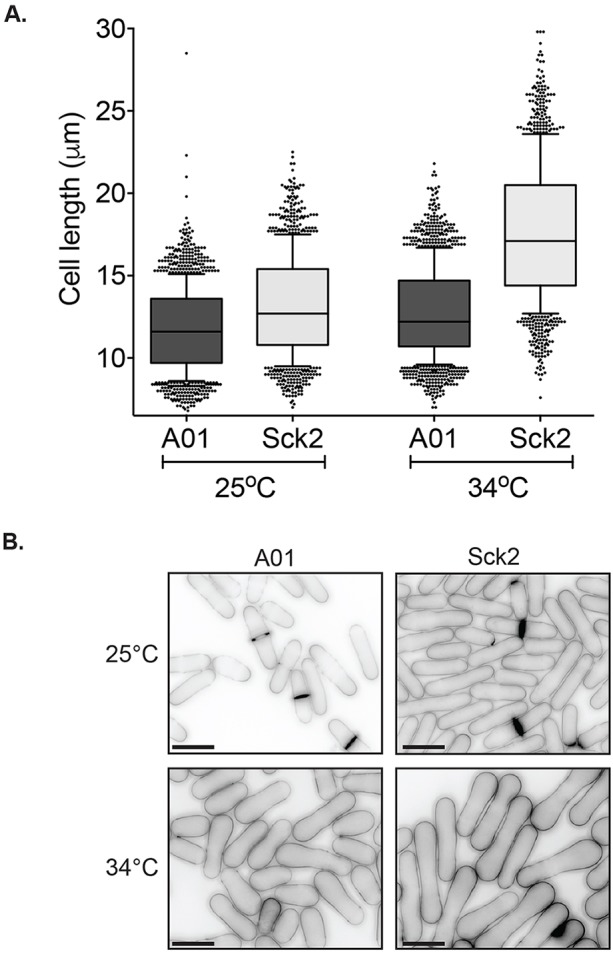
**Cell elongation in cells overexpressing Sck2.** (A) Cell length measurements from seven experiments were combined giving cell length values for >1000 cells per condition. In each experiment, control (A01) or Sck2-overexpressing (Sck2) cells were grown exponentially in EMM for 22 h and grown for a further 6 h at either 25°C or 34°C. Images were measured using the PointPicker plug-in of ImageJ (National Institutes of Health). Boxes are delimited by the first quartile, median and third quartile, and whiskers show the 10–90th percentiles. Values outside this range are displayed as individual dots. (B) Cells were grown exponentially in EMM for 22 h and imaged following growth for a further 6 h at either 25°C or 34°C. Scale bars: 10 μm.

The cell length increase caused by Sck2 overexpression in the *pat1-114 mei4Δ* strain at the restrictive temperature is shown in Fig. 3A,B, and Fig. S3A. To rule out the possibility that the effects of Sck2 overexpression were due to cells returning from a meiotic to a mitotic programme, we examined the expression of genes involved in meiosis (*bqt1^+^*, *mcp6^+^* and *mcp7^+^*) in both control and Sck2-overexpressing cells. The expression of these genes was induced by a similar amount in the control and Sck2-overexpressing strains at the restrictive temperature (Fig. S3B), indicating the Sck2 overexpressing cells were still within the meiotic developmental programme.

**Fig. 4.**
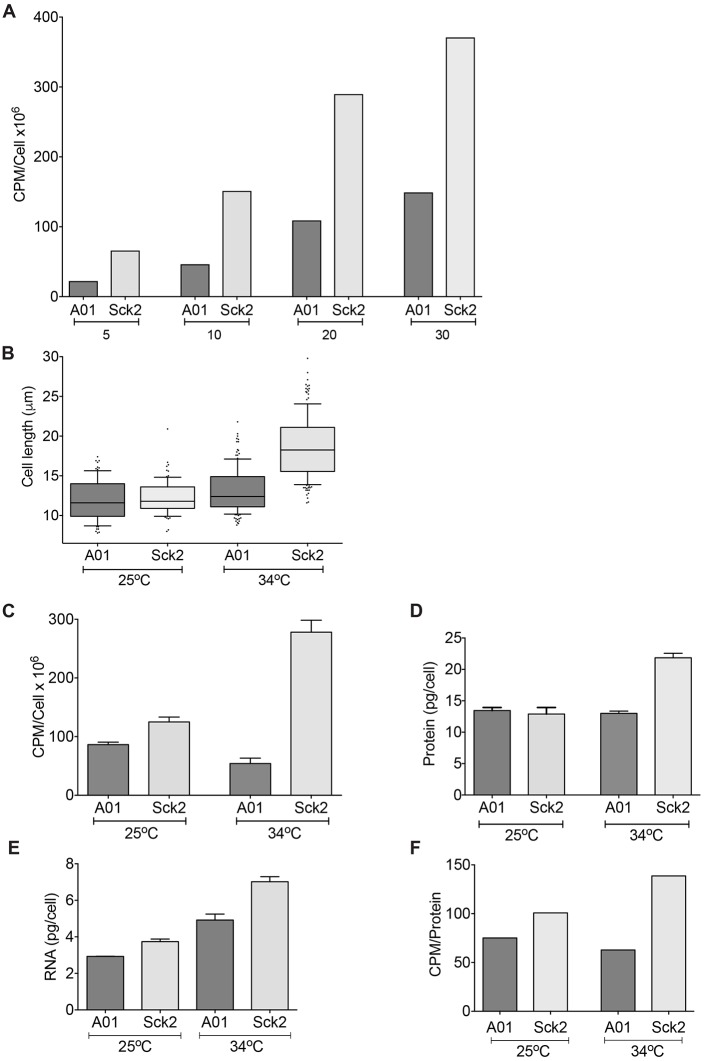
**Sck2 overexpression induces a transcription rate increase.** (A) Cells were grown for 22 h to induce expression of the control gene, A01, or *sck2^+^* (Sck2), then shifted to 34°C to activate the meiotic program. After 6 h at 34°C, [^3^H]adenine incorporation per cell was measured after 5, 10, 20 and 30 min of labelling. Technical duplicate samples were analysed and the mean is presented. (B) Cell length measurements. More than 100 cells per condition were analysed. Boxes are delimited by the first quartile, median and third quartile, and whiskers show the 10–90th percentiles. Values outside this range are displayed as individual dots. (C) [^3^H]adenine incorporation into total RNA per cell. Technical duplicate samples analysed. Error bars represent the s.e.m. (D) Protein content per cell. Technical triplicate samples analysed. Error bars represent the s.e.m. (E) RNA content per cell. Technical triplicate samples analysed. Error bars represent the s.e.m. (F) Mean [^3^H]adenine incorporation into total RNA normalised to mean cellular protein content.

Given that two of the cell growth-promoting genes were RNA polymerase subunits, we proceeded to test whether transcription could be involved in Sck2-induced continuation of cellular growth. We measured the transcription rate by assaying total RNA transcription through [^3^H]adenine pulse labelling using conditions that mostly reflect RNA synthesis rather than RNA turnover (Fraser, 1975). In meiotic arrest, the transcription rate was greater in Sck2-overexpressing cells than in the control strain (Fig. 4A), indicating that transcription is more active in the Sck2-overexpressing strain. Transcription rate normally varies with cell size and Zhurinsky et al. (2010) reported that cells within a 2-fold size-range of the wild type maintain similar transcription rates per µg protein (Zhurinsky et al., 2010). Sck2-overexpressing cells were 43% larger than control cells at 34°C (Fig. 4B), but the transcription rate was elevated 5-fold (Fig. 4C). The protein amount per cell increased 68% in Sck2-overexpressing cells (Fig. 4D) and total RNA levels were 43% higher in Sck2-overexpressing cells (Fig. 4E), but the transcription rate per protein was 3-fold higher than in control cells (Fig. 4F), indicating that the transcription rate was significantly induced by Sck2 overexpression. Additionally, we treated Sck2-overexpressing cells at 34°C with a transcription inhibitor, thiolutin or 1-10 phenanthroline, and assayed cellular growth. In the presence of either drug, cell lengthening was inhibited, indicating that transcription is required for Sck2-dependent cell growth (Fig. S4A). Together, these data suggest that Sck2 has a rate-limiting role for transcription, which in turn has a role in regulating cellular growth during meiotic growth arrest.

We next investigated whether other putative S6 kinases could initiate cell growth when overexpressed, and found that overexpression of Sck1 or Psk1 led to only a small increase in cell growth at 34°C and that overexpression of Gad8 had no effect (Fig. S5). This indicates that Sck2 is the most potent of the TORC1 effectors in promoting cell growth.

**Fig. 5.**
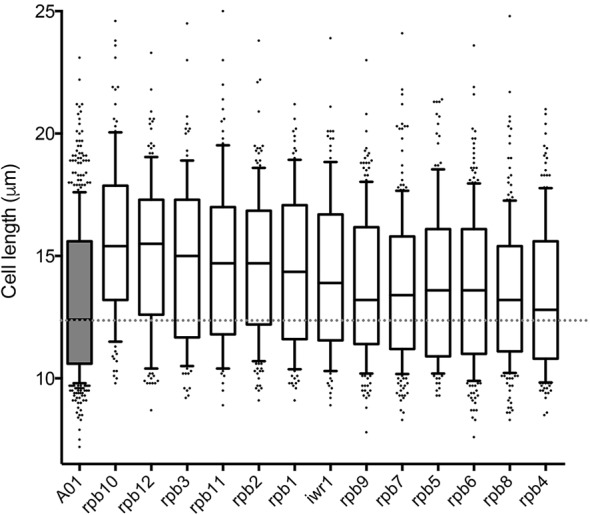
**RNA polymerase subunits promote cell length increase in *pat1-114 mei4Δ* cells at the restrictive temperature.** Cell length measurements for cells grown at 25°C for 22 h to induce expression of the indicated gene, then shifted to 34°C to induce meiotic entry. A01, control. Cell length after 6 h at restrictive temperature is shown. Boxes are delimited by the first quartile, median and third quartile, and show the 10–90th percentiles. Values outside this range are displayed as individual dots. *n*>100 cells per strain were analysed.

Given the transcription rate increase observed in Sck2-overexpressing cells, we re-examined our initial screen hits. As mentioned above, two of our eight final growth-promoting candidates were RNA polymerase (Rpb) subunits, Rpb11 and Rpb6. Additionally, among our second-tier candidates, which increased cell length by 8–30%, we found Rpb5 and Srb4, a subunit of the Mediator complex (a coactivator of RNA polymerase II-dependent transcription; Spahr et al., 2000). These observations prompted us to consider the role of other RNA polymerase subunits in our system. To this end, we integrated and overexpressed the 12 Rpb subunits, as well as Iwr1, which imports RNA polymerase II into the nucleus, into the *pat1-114 mei4Δ* strain. We noted cell length increases with all but Rpb8 and Rpb4 (Fig. 5), giving further support to the idea that control of RNA transcription regulates global cellular growth.

## DISCUSSION

This study has uncovered regulators of cell growth that are independent of nutrient availability. Eight genes were found which, when overexpressed, caused cells to grow significantly during meiotic arrest and are therefore potentially rate limiting for cellular growth.

The presence of genes involved in transcription (three DNA-directed RNA polymerase subunits and the RNA polymerase II transcriptional cofactor *srb4*) among our primary screen candidates suggests that the transcription rate is limiting for cell growth. Zhurinsky et al. (2010) proposed that there is a global cellular control that regulates the overall transcription rate in the cell and coordinates the transcription rates of the majority of genes (Zhurinsky et al., 2010). It was proposed that there is a factor (or factors) limiting gene transcription, whose level is determined by the protein content of the cell. Transcription machinery components, such as RNA polymerase, were suggested as potential candidates for this factor (Zhurinsky et al., 2010). Furthermore, RNA polymerase subunits Rpb1, Rpb2, Rpb3, Rpb6, Rpb7 and Rpb9 have all been reported to be haploinsufficient for cellular fitness (Kim et al., 2010), further suggesting that RNA polymerase subunits themselves might be limiting for cell growth.

The strongest cell elongation phenotype observed in the screen was due to overexpression of Sck2. As Sck2 is thought to act downstream of TORC1, a positive growth regulator, it is notable that we did not pick up Tor1 or Tor2 in our screen. This could be because the Tor pathway cannot be fully activated in our system by simple overexpression of Tor. We observed only moderate cell lengthening when Sck1 and Psk1, other TORC1 targets, were overexpressed, suggesting that Sck2 has a more prominent role in cellular growth control. We further demonstrate that Sck2 is rate limiting for transcription, because overexpression of Sck2 increases the transcription rate per protein in meiotic G2 arrest. The established role of budding yeast Sch9 in modulating RNA Pol II-dependent expression of ribosomal proteins, together with its role in Pol I- and Pol III-dependent transcription, positions Sch9 as a key coordinator of ribosome biogenesis and protein synthesis through transcription regulation (Jorgensen et al., 2004; Johnson, 2010; Du et al., 2012). Sck2 may be carrying out analogous functions to Sch9 by enhancing transcription, which subsequently stimulates ribosome biogenesis, and may facilitate the positive effect of Sck2 on cell growth. Sck2 has been previously placed in the Tor signalling pathway, but this is the first report showing that it can regulate overall cell growth.

We set out to identify factors involved in global cell growth regulation in a context where nutrients are not limiting and the cell cycle is arrested. Our finding that, in addition to Sck2, numerous identified genes are involved in transcription indicates that global regulation of transcription itself is a key step in cellular growth control. In a growing budding yeast cell, 60% of total transcription is devoted to ribosomal RNA and 50% of RNA polymerase II transcription is devoted to ribosomal protein genes (Warner, 1999). An increase in overall transcription would therefore favour an increase in the synthesis of ribosomal components, which would be necessary to promote an increase in cell mass through increased protein translation capacity. Our study has revealed a central role for global transcription mediated through S6 protein kinase signalling on cellular growth control.

## MATERIALS AND METHODS

### Strains and growth conditions

*S. pombe* media and methods used are described in Moreno et al. (1991). Strains used are listed in Table S2. Initial screening experiments were carried out in agar minimal medium supplemented with 0.15 mg/ml L-histidine, L-leucine and adenine (EMM-U) with 5 μg/ml thiamine (+T) or without (−T) at 25°C or 34°C. Phenotype confirmation experiments were carried out in liquid minimal medium supplemented with 0.15 mg/ml L-histidine, adenine and uridine (EMM-L) (+/−T) at 25°C or 34°C. RNA content, protein content and transcription rate experiments were carried out in minimal medium (EMM) without supplements at 25°C or 34°C.

### Screen for cell growth

The Riken ORFeome plasmid collection was transformed into *pat1-114 mei4::kan ura4-D18* strains and the resultant transformants screened for suppression of the growth arrest. A *pat1-114 mei4::kan ura4-D18* strain transformed with a plasmid containing the gene SPBC1711.15c (pDual-YFH1c-SPBC1711.15c) was used as a control strain (denoted A01). This gene is annotated as an uncharacterised *S. pombe*-specific protein, and its deletion has no obvious effect on cell size, growth or population doubling time (Kim et al., 2010). The 52 plates of recombinant bacteria from the Riken ORFeome collection were inoculated using a pin-tool into 45 plates (each of 96 wells with 2 ml capacity) containing 1 ml of LB medium with 50 μg/ml ampicillin, and incubated at 37°C for 16 h with shaking. Each plate was combined, and plasmids were prepared using the Qiaprep spin miniprep kit (Qiagen). Each pooled plasmid preparation was transformed into the *pat1-114 mei4::kan ura4-D18* strain and selected for the ability to grow on EMM-U+T at 25°C. For each transformation, 1000 colonies were patched on EMM-U+T at 25°C in order to represent 99% of the plasmids within the pool [probability assumed using formula described in Zilsel et al. (1992)]. Plates were replica plated to one EMM-U+T plate and two EMM-U-T plates. After 24 h growth, one of the EMM-U plates was moved to 34°C for a further 16 h. Transformants were then visually screened for those that had elongated on the plate at 34°C, but not on the plates at 25°C when compared with the control. The visual screen for cell size phenotypes was carried out using a Zeiss Axioskop 40 microscope equipped with a 20×0.4 NA objective and an additional 1.8× magnification. From this first screen, we selected ∼500 different transformed strains for a second screen in liquid culture, in which growth conditions are better controlled. Candidate transformants were grown as above but in individual flasks containing 15 ml of EMM-U–T medium at 25°C and 34°C, and cell size was screened following 16 h after the temperature shift to 34°C (and when the culture at 25°C was growing in exponential phase). The cell wall and septum were stained with Calcofluor (Sigma), and cells were observed with a Zeiss Axioskop microscope, equipped with a 63×1.4 NA objective and a Zeiss AxioCam MRm camera. Images were captured for transformed strains that appeared to be longer than the control strain and the length of arrested and dividing cells measured from captured images. A total of 40 transformed strains were confirmed to be elongated compared to the control strain.

For these strains, cells were grown in liquid culture and plasmids recovered using the QIAprep^®^ Spin Miniprep Kit (Qiagen, protocol adapted by Michael Jones, Chugai Institute for Molecular Medicine, Ibaraki, Japan). The recovered plasmid was sequenced using the following primer: 5′-GGAAGAGGAATCCTGGCA-3′. The location of the plasmid within the Riken library was also checked against the pool number used for the initial transformation. The plasmids identified were prepared individually from the Riken ORFeome, digested with the restriction enzyme *Not1* and integrated by transformation into the strain *pat1-114 mei4::nat ura4 D18 leu1-32* at the *leu1* locus, as described for the pDUAL vector series (Matsuyama et al., 2004). Candidate transformants were grown in individual flasks containing 15 ml of EMM-L at 25°C, and half the culture moved to 34°C for 6 h. Cell length measurements confirmed 24 candidate genes that cause cell elongation in the meiotic arrest without affecting cell length in mitotically dividing cells.

### Cell length measurements and statistical methods

Cell length was measured from pictures of live Calcofluor-stained cells by using the PointPicker plug-in of ImageJ (National Institutes of Health). Average cell length values were determined from at least 100 cells (whole imaged population). In box-and-whisker plots, boxes are delimited by the first quartile, median and third quartile, and whiskers mark data within the 10–90th percentiles. Values outside this range are displayed as individual dots.

### Transcription rate assays

For transcription rate analysis, 3.5 μCi of [^3^H]adenine (21 μCi/nmol; Amersham) and 7.4 μM unlabelled adenine were added to 1 ml culture aliquots grown in EMM, and samples were incubated for 10 min at the respective temperatures noted in the text and figures. The reaction was stopped by the addition of 10 ml ice-cold 10% trichloroacetic acid (TCA) containing 0.74 mM adenine. Precipitated RNA was collected by filtering through Durapore nitrocellulose filters (Millipore), followed by 6 washes with 10% TCA and one wash with ethanol. Filters were dried and their radioactivity determined in a LS6000IC scintillation counter (Beckman) by using UltimaGold scintillation liquid (PerkinElmer).

### Protein and RNA content

Cells were harvested by centrifugation (2200 ***g*** for 3 min), frozen in liquid nitrogen and washed twice in Milli-Q-H_2_O before determination of protein or RNA contents. Cellular protein contents were determined after overnight lysis in 1 M NaOH and 1% Triton X-100 by using a BioRad DC protein assay kit. Cellular RNA content was determined from the optical density (OD) at 260 nm following precipitation in 10% ice-cold TCA and two extractions in 5% TCA at 90°C for 15 min.

### Cell number measurements

Cell number was determined using a Sysmex XP-300.

### RNA isolation, RT-PCR and qPCR

RNA was isolated using hot phenol extraction (Schmitt et al., 1990). RNA was treated with RNase-free DNase and 1 μg RNA used as template in first-strand synthesis by Superscript III (Invitrogen) primed with oligo(dT)_20_. 1 μl cDNA was then used for quantitative PCR (qPCR) with the following primers: for *mcp6*, jg317, 5′-CAATCACTTTCACAGCAGTC-3′ and jg320, 5′-CAGGTTCAACGCATGAAACAG-3′; for *mcp7*, jg321, 5′-CGGAAAAGATTGGCACTAGC and jg324, 5′-GTTGCAGATCGTCCAAATCC-3′; for *sck2*, jg341, 5′-CGCTGATAACACACAACAAC and jg343, 5′-GTCCATATCGGAATTGTCC-3′; and for *bqt1*, jg305, 5′-CCCAAATCGCGATTTATACTC-3′ and jg308, 5′-CGTTTGACTAAAGGCACATGG-3′.

### Flow cytometry

DNA content per cell was determined from 10^4^ cells that were fixed with 70% (v/v) ethanol and then washed with 1 ml 50 mM sodium citrate. Cells were resuspended in 0.5 ml 50 mM sodium citrate containing 0.1 mg/ml RNase A and incubated at 37°C overnight. DNA was stained with 2 μg/ml propidium iodide and samples were sonicated before analysis in a BD FACSCalibur instrument.
